# Understanding informal payments in the health sector in Malawi: results of a multidistrict household survey

**DOI:** 10.1093/heapol/czag060

**Published:** 2026-05-08

**Authors:** Eric Umar, Maryam Chilumpha, Gertrude Chatha, Martin McKee, Blake Angell, Lucie Sabin, Dina Balabanova

**Affiliations:** Kamuzu University of Health Sciences (KUHeS), Department of Health Systems and Policy, Blantyre, Malawi; Kamuzu University of Health Sciences (KUHeS), Department of Health Systems and Policy, Blantyre, Malawi; Kamuzu University of Health Sciences (KUHeS), Department of Health Systems and Policy, Blantyre, Malawi; LSHTM, Department of Global Health and Development, WC1H 9SH, London, United Kingdom; George Institute for Global Health, Centre for Health Systems Science, Newtown, Australia; UCL Institute for Global Health, WC1N 1EH, London, United Kingdom; LSHTM, Department of Global Health and Development, WC1H 9SH, London, United Kingdom; LSHTM, Department of Global Health and Development, WC1H 9SH, London, United Kingdom

**Keywords:** Malawi, informal payments, corruption, health financing, equity, health systems

## Abstract

In Malawi, health services are officially free at the point of use, but patients often make informal payments to access services or obtain medicines. These payments undermine equity and trust in the health system. This study examined for the first time the prevalence, types, and determinants of informal payments among Malawians who had recently used health services. We conducted a multidistrict cross-sectional household survey in four districts chosen to reflect urban and rural Malawi. Households containing someone who had been hospitalized in the previous 6 months were interviewed using a structured questionnaire. Descriptive analyses identified the types and prevalence of informal payment, while multivariable logistic regressions identified factors associated with informal payments. Overall, 17% of respondents reported paying for services that are officially free. Informal payments are most often for medicines (52%), consumables (24%), and consultations (24%). Female respondents and those living in urban areas were significantly more likely to report giving informal payments, while those with higher levels of education were less likely. Participants with a good or very good financial situation were more likely to make such payments. Significant geographical variations were observed, with higher probabilities in Mchinji and Mzimba than in the Blantyre district. Nearly one in five Malawians reported making informal payments to access health services, questioning the formal policy that these are free. These findings highlight the need for governance reforms, greater accountability, and community awareness to reduce informal payments and promote equitable access to care.

Key messages

- Despite Malawi's policy of free healthcare at the point of use, informal payments remain widespread, with nearly one in five households reporting that they pay for services that are supposed to be free.
- Informal payments are most often made for medicines and consultations and are more likely among women, urban residents, and people who are better off financially.
- These practices undermine equity and trust in the health system, disproportionately disadvantage the poorest households, and may deter people from seeking care.
- To combat informal payments, governance must be reformed, accountability mechanisms strengthened, and public awareness of the right to free healthcare raised.

## Background

Informal payments in healthcare systems are widespread, particularly in low- and middle-income countries (LMICs) (Kabia et al. 2021). Informal payments involve unofficial transactions, such as bribes, gifts, or other financial inducements, often in response to inefficiencies or inadequacies within the formal healthcare system (Jahangiri and Aryankhesal 2017). The impact of informal payments is significant, leading to disparities in access and quality of care (Masiye et al. 2020, Kabia et al. 2021). Patients unable to afford these payments may receive inferior care or be excluded from essential services (Ensor 2004). Additionally, those who do not make informal payments may experience discrimination or neglect, reinforcing existing inequalities (Mtuwa and Chiweza 2023). Informal payments also skew the patterns of service delivery, as patients who pay may receive preferential treatment, affecting overall quality of care and fairness (Mphande-Namangale and Kazanga-Chiumia 2021).

Addressing informal payments is essential to restoring public trust in the medical profession and preventing additional costs to patients, particularly vulnerable groups (Khodamoradi et al. 2018). Various efforts have been undertaken to curb informal payments. There is now considerable evidence that mere top-down enforcement of formal rules and measures to increase transparency will be inadequate, on their own, in curbing informal payments, especially when powerful actors can circumvent them (Vian 2020, Ouma et al. 2024). Also, conventional measures to reduce informal payments can create perverse incentives for some actors, potentially reinforcing existing power imbalances (Liu et al. 2020). To effectively reduce informal payments, interventions should be tailored to specific risk factors, focus on prompt delivery of entitlements and benefits, and ensure ongoing oversight (Zandian et al. 2019).

A range of strategies has been proposed to tackle the persistent issue of informal payments in healthcare. One approach focuses on improving healthcare workers’ financial status by increasing their salaries. The rationale is that fair and adequate compensation can reduce the need for unofficial income sources and improve service delivery. Another strategy involves strengthening the regulatory framework, by enforcing stricter rules and enhancing transparency and accountability, health systems can ensure that all transactions are properly documented and monitored (Habib et al. 2017, Del Rey-Puech et al. 2025). Informing and empowering patients can also play a crucial role. Educating them about their rights and the formal procedures for accessing care enables them to advocate for equitable treatment and navigate the system more confidently (Mphande-Namangale and Kazanga-Chiumia 2021). In addition, creating confidential channels for reporting informal payments can help uncover hidden practices and prompt corrective action within the system. Finally, improving the operational capacity of health facilities, through better supply chain management and sufficient resource allocation, can reduce the systemic pressures that often give rise to informal payments (Doshmangir et al. 2020, Naher et al. 2020).

The Malawi national healthcare system is designed to provide free services at the point of delivery, funded by the government. Despite this, studies conducted in health facilities suggest that patients informally healthcare providers pay in money or in kind to secure faster treatment, access scarce medications, or obtain necessary surgical services (Bissell et al. 2018). The literature attributes these practices to systemic issues, such as inadequate funding and low salaries for healthcare workers, compelling them to seek additional income through unofficial channels (Masiye et al. 2020). Such practices are reported to pose significant barriers to accessing quality healthcare, particularly for economically disadvantaged populations. Despite the multiple existing complaint mechanisms in Malawi, these are little known and underused, which hampers the reporting of informal payments (Chilumpha et al. 2023).

Existing research on informal payments in Malawi has been limited in scope and methodology. Most studies have relied on exit interviews conducted within health facilities, which, while useful, offer only a partial view of the problem. These facility-based approaches tend to capture experiences of individuals who have successfully accessed care, potentially overlooking those who were deterred by informal payment demands or who sought care elsewhere. To gain a fuller understanding of the issue, a multidistrict cross-sectional survey was conducted across Malawi’s three regions. This broader approach aimed to establish the true prevalence of informal payments within the health system, explore their nature, and identify the factors that contribute to their occurrence.

## Methods

### Research questions

We sought to determine the prevalence of informal payments, to understand their nature, and identify the factors associated with their occurrence.

### Study design

We conducted a cross-sectional, multidistrict household survey in Malawi. Malawi is a landlocked country in southeastern Africa, bordered by Tanzania, Mozambique, and Zambia, with an estimated population of about 20 million. The population is predominantly young, with over half of the population under 18 years old, and largely rural, with around 80% of inhabitants residing in rural areas (UNICEF 2025). Malawi is classified as a low-income country, with a predominantly agrarian economy that is highly vulnerable to climate shocks, commodity price fluctuations, and dependence on external aid (World Bank 2025). Geographically, the country is characterized by Lake Malawi, which covers about one-fifth of its territory and is an important ecological and economic resource. Administratively, Malawi is divided into three regions, Northern, Central, and Southern, which are themselves subdivided into 29 districts. Although governance is officially decentralized through district councils, fiscal and administrative authority remains largely centralized.

Data were collected between August 2022 and February 2023 across Malawi's three regions: Southern, Central, and Northern. We selected one district in the Northern and Southern regions and two districts in the Central region to capture different contexts: Lilongwe (1.64 million residents, predominantly urban, capital city, hosting tertiary referral services and a mix of public and private health facilities, in the Central Region), Mchinji (0.60 million residents, predominantly rural, border district with Zambia, characterized by primary-level public health facilities and cross-border population movement, in the Central Region), Mzimba (0.94 million residents, predominantly rural, large inland district with dispersed populations and mainly public primary and secondary health services, in the Northern Region), and Blantyre (1.25 million residents, predominantly urban, major commercial and industrial hub, hosting tertiary referral services and a mix of public and private health facilities, in the Southern Region). Districts were purposively selected to capture variation in geographic location, urban-rural settings, and health system contexts across the country.

### Sampling

Households reporting a member hospitalized in the previous 6 months were invited to participate, resulting in a total of 846 participants interviewed (Cochran 1977). A minimum survey sample size of 800 was determined using the Cochran formula, which is appropriate for large populations such as Malawi.

We used a multistage cluster sampling: in the first stage, villages within selected traditional authorities were randomly chosen. In the second stage, within each village, research assistants started at the village headman’s house and selected every fourth household to the left, continuing this pattern throughout the village. In order to respect the principle of selecting one in four households while taking into account variations in village layout, the approach was adapted pragmatically. In villages where households were organized along paths or in a semi-linear layout, data collectors followed a clearly defined route (e.g. a main road encircling or crossing the village) and selected one in four households encountered along this route. In villages with more irregular or scattered layouts, where linear or grid progression was not possible, the ‘next’ household was defined as the nearest neighbouring dwelling in a consistent direction (e.g. always clockwise or always to the left). The interval of four was applied systematically in all cases to maintain consistency in the selection of households.

### Data collection

Data were collected as part of the Accountability in Action project. A research project conducted in Malawi and Nigeria and funded by the Medical Research Council to investigate the nature of corruption in health systems and explore potential intervention strategies to reduce it. More details on the study design are available elsewhere (Chilumpha et al. 2023).

We developed a survey questionnaire to capture the information needed to answer the research questions. Twenty-four pilot interviews were conducted in rural and urban areas of Blantyre to test the clarity of the questions and to identify potential skip patterns.

For the purposes of this study, informal payments were defined as any unofficial or unreceipted payments made by patients or their families to healthcare providers for services that are officially free. Examples provided to respondents included cash payments for medicines, gifts to jump the queue, or fees for consultations not recorded by the facility. These examples were included in the questionnaire to help clarify the distinction between informal payments and legitimate charges. Data were collected using computer-assisted personal interviews. Supervisors conducted random checks to ensure adherence to protocols.

Research assistants collecting data received 4 days of training, including the pilot phase. To reduce potential bias, data collectors were trained to explain the confidentiality of responses and to clarify that informal payments were being measured for research purposes only. Completed questionnaires were reviewed and approved by a supervisor before being securely transmitted to a password-protected cloud server.

### Analysis

We performed descriptive analyses to summarize respondent characteristics and the nature and distribution of informal payments using frequencies and proportions. We conducted a multivariable logistic regression to identify factors associated with the likelihood of making informal payments. Robust standard errors were pooled at the district level to account for intra-district correlation. All analyses were performed using Stata version 18.

We selected the independent variables for our multivariable model based on evidence from prior research showing that sociodemographic characteristics and individual perceptions are associated with the likelihood of making informal payments (Table 1). Studies have found that gender (Rajan et al. 2022), residential location, education, and household socioeconomic status are linked to variations in informal payment behaviour (Pourtaleb et al. 2020, Kabia et al. 2021, Nwokolo et al. 2025). Research also indicates that household size can influence exposure to and engagement in informal payments (Nwokolo et al. 2025). Perceptions and beliefs about informal payments are similarly identified in the literature as important behavioural drivers shaping whether patients engage in such practices (Pourtaleb et al. 2020).

**Table 1:** Description of the variables used in the model.

Name	Type	Description	Reference category
**Dependent variables**			
Paid an informal payment	Binary	Indicates whether the participant reported paying any informal payment for health services received in the past 6 months (1 = yes, 0 = no)	No
**Predisposing characteristics**			
Gender	Binary	Gender of the respondent, recorded as male or female	Male
Geographic location	Categorical	Place of residence of the household, classified as rural, urban, or peri-urban	Rural
Household size	Continuous	Number of individuals living in the household	–
Years of education	Continuous	Total number of years of formal schooling completed	–
Currently married	Binary	Indicate whether the respondent was currently married or living with a spouse at the time of the survey	No
Self-perceived financial situation	Categorical	Subjective assessment of the household’s financial situation, categorized as bad/very bad, average, or good/very good	Bad/very bad
Role in the household	Categorical	Position in the household, categorized as female head, male head, wife, husband, or other household member	Female head
District	Categorical	District of residence, categorized as Blantyre, Lilongwe, Mchinji, or Mzimba	Blantyre
Expect to pay extra	Categorical	Opinion on the need to pay additional fees for health services, categorized as agree, disagree, or not specified	Agree
Must pay for good quality care	Categorical	Perception of whether paying extra is necessary to receive good quality care, categorized as agree, disagree, or not specified	Agree
Right for the health worker to demand extra	Categorical	Belief about whether it is acceptable for health workers to demand extra payments, categorized as agree, disagree, or not specified	Agree

## Results

### Descriptive statistics

Table 2 presents the demographic characteristics of the 846 participants from households reporting a hospitalization in the previous 6 months. Most participants were female and lived in rural areas. The majority of participants were from Lilongwe district, and more than half belonged to households of four to six people. Regarding education, the majority had completed primary school. Most participants lived with a spouse and considered themselves to be in a very poor or poor financial situation.

**Table 2:** Demographic characteristics of participants.

Variable	Categories	Frequency^a^	Paid informal payment^b^
Gender	Male	199 (25.3)	29 (20.1)
	Female	586 (74.7)	114 (79.2)
Geographic location	Rural	444 (56.4)	69 (47.9)
	Urban	316 (40.1)	72 (50.0)
	Peri-urban	28 (3.5)	3 (2.1)
District	Blantyre	202 (25.6)	35 (24.3)
	Lilongwe	360 (35.7)	59 (41.0)
	Mchinji	84 (10.7)	68 (47.2)
	Mzimba	142 (18.0)	108 (75.0)
Household size	1–3	216 (27.4)	37 (25.7)
	4–6	444 (56.4)	85 (59.0)
	6–9	114 (14.5)	20 (13.9)
	10+	12 (1.7)	2 (1.4)
Highest education level	No school	62 (7.9)	6 (4.2)
	Primary	401 (50.9)	77 (53.5)
	Secondary	309 (39.2)	57 (39.6)
	University	16 (2.0)	4 (2.8)
Marital status	Single	48 (6.1)	7 (4.9)
	Living with a spouse	564 (71.8)	102 (70.8)
	Widowed	80 (10.2)	17 (11.8)
	Divorced/separated	94 (11.9)	18 (12.5)
Self-perceived financial situation	Very good/good	83 (10.6)	22 (15.3)
	Average	261 (33.3)	48 (33.3)
	Very bad/bad	434 (55.4)	74 (51.4)
	Not specified	6 (0.7)	0 (0.0)
Expect to pay extra	Agree	131 (16.7)	41 (28.5)
	Disagree	613 (78.2)	98 (68.1)
	Not specified	40 (5.1)	4 (2.8)
Must pay for good quality care	Agree	168 (21.5)	47 (32.6)
	Disagree	545 (69.7)	84 (58.3)
	Not specified	69 (8.8)	13 (9.0)
Right for the health worker to demand extra	Agree	252 (33.0)	60 (41.7)
	Disagree	459 (58.6)	72 (50.0)
	Not specified	66 (8.4)	12 (8.3)

^a^Percentage of the total population in parentheses in the column.

^b^Percentage of respondents in each group responding ‘yes’ to whether they paid informal payments of any kind in parentheses in the column (*n* = 144).

Participants’ perceptions of informal payments vary depending on the statements. Approximately 16.7% of respondents agreed that it was expected in Malawi to pay extra to access health services, and 33.0% agreed that it was acceptable for health workers to demand additional payment. When asked whether it was necessary to pay for good-quality care, 21.5% responded affirmatively.

Overall, 144 participants (17.0% of the sample) reported making informal payments for health services that were officially supposed to be free. The proportion of participants reporting informal payments varied across sociodemographic groups, with the highest percentages observed among females, those living in urban areas, and those living with their spouse. Informal payments were more common in households with four to six members and among participants with primary education. Regarding perceived financial situation, participants reporting a very poor or poor situation accounted for 51.4% of informal payments.

Table 3 presents the types of services for which respondents reported making informal payments for services that were officially supposed to be free. The most commonly reported payments were for drugs, followed by consumables and consultation.

**Table 3:** Types of health services for which respondents reported making informal payments.

Type of service	Frequency
Drugs	75 (52.1)
Consumables	34 (23.6)
Consultation	34 (23.6)
Health certificate	20 (13.9)
Diagnostic tests	16 (11.1)
To jump the queue	11 (7.6)
Unspecified service	13 (9.0)

Percentages presented in parentheses were based on respondents (*n* = 144) who reported making informal payments for services that were supposed to be free of charge.

### Econometric analysis

Table 4 presents the adjusted odds ratios for factors associated with the likelihood of making informal payments. Females were significantly more likely than males to report informal payments. Participants living in urban areas were nearly twice as likely to report informal payments as those living in rural areas. Each additional year of education was significantly associated with a slightly lower probability of informal payments. Respondents who reported good or very good financial status were significantly more than twice as likely to make informal payments as those who reported poor or very poor financial status. Regarding household roles, male heads of household and wives were significantly less likely than female heads of household to report informal payments. Participants from Mchinji and Mzimba were significantly more likely to report informal payments than those from Blantyre. Perceptions regarding paying extra for care and quality, or the right of health workers to demand additional payments, were not significantly associated with the likelihood of informal payments.

**Table 4:** Adjusted odds ratio of factors associated with the likelihood of paying informal payments.

Variable	Odds ratio	95% CI
Female	1.25***	1.14 ; 1.36
Geographic location		
Peri-urban	0.45	0.11 ; 1.79
Urban	1.90**	1.02 ; 3.54
Household size	0.95	0.89 ; 1.01
Years of education	0.96*	0.92 ; 1.00
Currently married	1.44	0.86 ; 2.40
Self-perceived financial situation		
Average	1.28	0.62 ; 2.66
Good/very good	2.13**	1.09 ; 4.18
Role in the household		
Male head	0.35**	0.14 ; 0.85
Wife	0.48**	0.27 ; 0.86
Husband	0.78	0.14 ; 4.27
Other	0.77**	0.57 ; 1.03
District		
Lilongwe	1.03	0.93 ; 1.13
Mchinji	1.65***	1.21 ; 2.25
Mzimba	2.06***	1.59 ; 2.66
Expect to pay extra		
Disagree	0.46	0.15 ; 1.42
Not specified	0.20**	0.06 ; 0.71
Must pay for good quality care		
Disagree	0.66	0.20 ; 2.26
Not specified	1.26	0.60 ; 2.62
Right for the health worker to demand extra		
Disagree	1.17	0.57 ; 2.41
Not specified	1.12	0.39 ; 3.25
Observations	777	
R-squared	0.07	

* *P* < 0.10, ** *P* < 0.05, *** *P* < 0.01. CI, confidence interval.

## Discussion

To the best of our knowledge, this is the first study of the prevalence of informal payments using consistent methods across the three regions of Malawi. We found that 17.0% of households that included someone who had sought care had paid at the health facility for services that were supposed to be free. Informal payments were most common for medicines, followed by consumables and consultations. Women, urban residents, and those in better financial situations were more likely to make informal payments. At the same time, higher levels of education and being a male head of household or wife were associated with lower probabilities. Significant geographical differences were also observed between districts. Overall, these findings were consistent with those from a global systematic literature review of factors associated with informal payments (Pourtaleb et al. 2020). These associations should be interpreted with caution. They may reflect differences in healthcare utilization patterns, exposure to providers, household decision-making dynamics, and ability to pay rather than direct causal relationships. In addition, unmeasured factors, such as the type of facility accessed, frequency of healthcare use, and local health system conditions, may also influence these patterns.

Although the prevalence of informal payments in this study is high (17%), it is lower than that reported in a previous study conducted in a central hospital in Malawi, where ∼47% of respondents admitted to making informal payments to access health services (Mphande-Namangale and Kazanga-Chiumia 2021). This difference likely reflects the larger and more diverse sample in the current survey, which included participants from various districts, rather than participants from a district recruited through a single tertiary hospital. The persistence of informal payments has important implications for Malawi’s progress towards Universal Health Coverage (UHC). UHC, as defined by the World Health Organization, requires that all individuals access needed services without financial hardship (Mchenga et al. 2022). The finding that 17% of respondents paid for services that are officially free indicates the presence of hidden out-of-pocket costs. Informal payments also threaten equity, as better-off households would be able to secure services through additional informal payments, potentially creating unequal access within public health facilities. Addressing informal payments is therefore essential for advancing equitable and effective UHC in Malawi.

A significant proportion of respondents (17.0%) agreed with the statement that ‘Malawians expect to pay something extra when seeking healthcare’, demonstrating the deep embedding of informal payments in Malawi's health system. This perception aligns with the findings of a previous study conducted in a tertiary hospital in Malawi, where 80% of respondents reported being aware of informal payments for accessing health services (Mphande-Namangale and Kazanga-Chiumia 2021). This normalization of informal payments highlighted systemic governance issues in the health system (Masefield et al. 2020) and persistent inequalities in access to health services in Malawi (Mulaga et al. 2022).

Contrary to the findings in Malawi, a study on medical corruption in China, based on judicial data, revealed that corruption mainly took the form of bribes in the health sector. Although healthcare providers were also the main beneficiaries of these bribes, the sources differed: in China, payments primarily came from suppliers of pharmaceuticals and medical equipment, whereas in Malawi, informal payments were more often initiated by patients seeking services. The study also reported relatively low levels of informal payments made by patients in China compared to Malawi and other LMICs, highlighting important contextual differences in how corruption manifests itself in health systems (Fu et al. 2023).

We found that females were more likely than males to report making informal payments for services that were supposed to be free. This finding is consistent with that of Mphande-Namangale and Kazanga-Chiumia (2021) and may reflect women's greater contact with health services as they seek health services, particularly maternal and child health services. Power imbalances between males and females within the health system in Malawi, particularly in doctor–patient interactions, may also increase female patients’ vulnerability to informal payment requests (Azad et al. 2020).

Individuals living in urban areas were more likely to report informal payments than those living in rural areas. This may be explained by the greater demand for services in densely populated urban areas, coupled with constrained health system capacity. Together, these pressures may lead individuals to use any available means, including informal payment, to secure access to limited health resources. Further, demand may also be higher because healthcare services in urban areas are often perceived as offering better quality of care (Collins et al. 2025). In urban areas where patient numbers are high and resources are limited (McBride and Moucheraud 2022), patients may be more inclined to offer informal payments to obtain faster and better care. Furthermore, as urban residents tend to be relatively better off financially, they may be able and willing to make informal payments to obtain faster or better quality services.

Participants from the districts of Mchinji and Mzimba were significantly more likely to report informal payments than those from Blantyre. These regional differences may reflect variations in governance, health facility management and access, and local accountability mechanisms (Masefield et al. 2020).

We also found that financially better-off respondents were more likely to make informal payments, suggesting that the ability to pay facilitates such transactions. This is likely to exacerbate inequalities in access to health services between those who cannot afford to pay for services that should be free and those who can (Schaaf and Topp 2019, Mphande-Namangale and Kazanga-Chiumia 2021). Conversely, higher levels of education were associated with a lower likelihood of making informal payments. More educated individuals may be better informed about their rights to free care or more confident in their ability to refuse such payments. This is consistent with the findings of Mphande-Namangale and Kazanga-Chiumia (2021), who reported that many patients in public hospitals in Malawi were unaware of their rights, which limited their ability to question requests for informal payments.

This study has several limitations. For logistical and financial reasons, it was only possible to include four of Malawi’s 28 districts, although from the three regions of the country. This potentially excluded areas with different health system dynamics, governance structures, or socioeconomic conditions. The inclusion criteria focused solely on households with a member recently hospitalized, which may have overlooked individuals who accessed outpatient services or avoided care altogether due to informal payment demands. Additionally, the sampling method, starting from the village headman’s house and selecting every fourth household, may have introduced spatial bias, particularly in communities with uneven household distribution. The reliance on self-reported data also poses a risk of underreporting, as informal payments are sensitive and may be misunderstood or deliberately concealed by respondents. Finally, while both urban and rural areas were included, differences in sample size and contextual factors such as service availability and patient expectations may have skewed the results towards more accessible or better-resourced settings. Analytically, the sampling may have led to an underestimation of standard errors, warranting caution in interpreting district-level effects. Furthermore, the cross-sectional design limits the ability to infer causality, and the observed associations may also reflect unmeasured confounders such as the type of facility accessed, frequency of service use, or differences in healthcare utilization patterns. Nevertheless, this study is the first multidistrict cross-sectional study of informal payments in the Malawian health system, providing new empirical evidence to the limited literature on the determinants of informal payments in low-income settings. Further qualitative research exploring the experiences of individuals who have made informal payments could complement this study and provide deeper insight into the contextual, social, and systemic factors that sustain these practices, while shedding light on variations across districts and household socioeconomic groups.

## Conclusion

This study provided the first multidistrict cross-sectional data on the extent and determinants of informal payments in Malawi's public health system. Nearly one in five respondents reported paying for services that should have been free, with some users, such as women, particularly affected, questioning the right to access health services for free and perpetuating inequalities in access. To combat informal payments, law enforcement alone is not enough. Targeted reforms should include improving health worker remuneration to reduce financial incentives for informal payments, establishing community-based oversight mechanisms to enhance accountability, and launching public education campaigns to inform patients of their rights and entitlements and empower them to challenge informal payments. These measures, tailored to Malawi’s health system context, are essential for promoting equity, restoring trust, and advancing progress towards universal health coverage.

However, the findings should be interpreted in light of the study’s limitations, including the inclusion of only four of Malawi’s 28 districts, focus on recently hospitalized households, reliance on self-reported data, and potential spatial and sampling biases, which may limit the generalisability of the results and limit causal inference. Further research using mixed-methods, combining quantitative surveys with qualitative approaches, is needed to capture both the prevalence and the nuanced contextual drivers of informal payments in Malawi.

## Data Availability

The data underlying this article will be shared on reasonable request to the corresponding author

## Reflexivity statement

This study was conducted as part of an international collaboration between Malawian, Nigerian, and British researchers. The research team included authors based in Malawi with direct knowledge of the local health system, as well as international collaborators specializing in health system governance and health financing in low-income countries. This diversity of perspectives ensured that the study design, data collection, and interpretation were contextually grounded while maintaining analytical rigour. Throughout the research process, the team was conscious of its position, recognizing that differences in context, disciplinary orientation and institutional affiliation could influence how informal payments were perceived and their implications for the equity of the health system in Malawi.

## References

[czag060-B1] Azad AD, Charles AG, Ding Q et al The gender gap and healthcare: associations between gender roles and factors affecting healthcare access in Central Malawi, June–August 2017. Arch Public Health 2020;78:119. 10.1186/s13690-020-00497-w 33292511 PMC7672876

[czag060-B2] Bissell P, Pienaar E, Chirwa L. Informal payments and their impact on healthcare delivery in Malawi. Health Policy Plan 2018;33:565–73. 10.1093/heapol/czag060

[czag060-B3] Chilumpha M, Chatha G, Umar E et al “We stay silent and keep it in our hearts”: a qualitative study of failure of complaints mechanisms in Malawi’s health system. Health Policy Plan 2023;38:ii14–24. 10.1093/heapol/czad043 37995264 PMC10666912

[czag060-B4] Cochran WG . Sampling Techniques. New York: John Wiley & Sons, 1977.

[czag060-B5] Collins JH, Tafesse W, Chitsulo P et al Healthcare service user-reported quality of care in Malawi: a national multi-facility cross-sectional study. medRxiv, pp. 2025–09. 2025. 10.1136/bmjopen-2025-115140 PMC13331198 42379724

[czag060-B6] Del Rey-Puech P, Balabanova D, McKee M. Artificial intelligence and corruption: opportunities and challenges in the health sector. Int J Health Plann Manage 2025;40:1341–7. 10.1002/hpm.70002 40498655 PMC12579520

[czag060-B7] Doshmangir L, Sajadi HS, Ghiasipour M et al Correction to: Informal payments for inpatient health care in post-health transformation plan period: evidence from Iran. BMC Public Health 2020;20:781. 10.1186/s12889-020-08924-x 32450836 PMC7249408

[czag060-B8] Ensor T . Informal payments for health care in transition economies. Soc Sci Med 2004;58:237–46. 10.1016/S0277-9536(03)00007-8 14604610

[czag060-B9] Fu H, Lai Y, Li Y et al Understanding medical corruption in China: a mixed-methods study. Health Policy Plan 2023;38:496–508. 10.1093/heapol/czad015 36798965

[czag060-B10] Habib MA, Raynes-Greenow C, Nausheen S et al Prevalence and determinants of unintended pregnancies amongst women attending antenatal clinics in Pakistan. BMC Pregnancy Childbirth 2017;17:1–10. 10.1186/s12884-017-1339-z 28558671 PMC5450067

[czag060-B11] Jahangiri R, Aryankhesal A. Factors influencing on informal payments in healthcare systems: a systematic review. Med Ethics J 2017;11:73–92. 10.21859/mej-114073

[czag060-B12] Kabia E, Goodman C, Balabanova D et al The hidden financial burden of healthcare: a systematic literature review of informal payments in Sub-Saharan Africa. Wellcome Open Res 2021;6:297. 10.12688/wellcomeopenres.17228.1 36199622 PMC9513412

[czag060-B13] Khodamoradi A, Rashidian A, Daryabeygi-Khotbehsara R et al Evaluation of informal payments to health care professionals and the influential factors in Urmia city hospitals, Iran. J Med Ethics Hist Med 2018;11:7. PMCID: PMC6642461, PMID: 31346384. 31346384 PMC6642461

[czag060-B14] Liu N, Bao G, He AJ. Does health insurance coverage reduce informal payments? Evidence from the “red envelopes” in China. BMC Health Serv Res 2020;20:95. 10.1186/s12913-020-4955-7 32028953 PMC7006416

[czag060-B15] Masefield SC, Msosa A, Grugel J. Challenges to effective governance in a low income healthcare system: a qualitative study of stakeholder perceptions in Malawi. BMC Health Serv Res 2020;20:1142. 10.1186/s12913-020-06002-x 33317520 PMC7734892

[czag060-B16] Masiye F, Kaonga O, Banda CM. Informal payments for primary health services in Zambia: evidence from a health facility patient exit survey. Health Policy OPEN 2020;1:. 10.1016/j.hpopen.2020.100020 PMC10297501 37383307

[czag060-B17] McBride K, Moucheraud C. Rural–urban differences: using finer geographic classifications to reevaluate distance and choice of health services in Malawi. Health Syst Reform 2022;8:e2051229. 10.1080/23288604.2022.2051229 35416748 PMC9995164

[czag060-B18] Mchenga M, Manthalu G, Chingwanda A et al Developing Malawi’s universal health coverage Index. Front Health Serv 2022;1:786186. 10.3389/frhs.2021.786186 36926481 PMC10012749

[czag060-B19] Mphande-Namangale A, Kazanga-Chiumia I. Informal payments in public hospitals in Malawi: the case of Kamuzu Central Hospital. Glob Health Res Policy 2021;6:41. 10.1186/s41256-021-00225-z 34814949 PMC8611973

[czag060-B20] Mtuwa S, Chiweza AL. Implications of corruption on Public Administration in Malawi. J Humanit 2023;31:91–112. 10.4314/jh.v31i1.5

[czag060-B21] Mulaga AN, Kamndaya MS, Masangwi SJ. Decomposing socio-economic inequality in catastrophic out-of-pocket health expenditures in Malawi. PLOS Glob Public Health 2022;2:e0000182. 10.1371/journal.pgph.0000182 36962147 PMC10021269

[czag060-B22] Naher N, Hoque R, Hassan M. The influence of corruption and governance in the delivery of frontline health care services in the public sector: a scoping review of current and future prospects in low and middle-income countries of south and south-east Asia. BMC Public Health 2020;20:880. 10.1186/s12889-020-08975-0 32513131 PMC7278189

[czag060-B23] Nwokolo C, Onwujekwe O, McKee M et al Household health-seeking behaviour and response to Informal payment: does economic status matter? Health Econ Rev 2025;15:83. 10.1186/s13561-025-00654-3 41099997 PMC12532436

[czag060-B24] Ouma S, Beltrame DC, Mitlin D et al Informal settlements: Domain report. ACRC Working Paper, African Cities Research Consortium: The University of Manchester. 2024. www.african-cities.org

[czag060-B25] Pourtaleb A, Jafari M, Seyedin H et al New insight into the informal patients’ payments on the evidence of literature: a systematic review study. BMC Health Serv Res 2020;20:14. 10.1186/s12913-019-4647-3 31902368 PMC6943960

[czag060-B26] Rajan S, Santoso C, Abba-Aji M et al Gender differences in informal payments for healthcare: evidence from 34 African countries. Health Policy Plan 2022;37:132–9. 10.1093/heapol/czab123 34662388

[czag060-B27] Schaaf M, Topp SM. A critical interpretive synthesis of informal payments in maternal health care. Health Policy Plan 2019;34:216–29. 10.1093/heapol/czz003 30903167 PMC6528746

[czag060-B28] UNICEF Executive Board . *Malawi Country Programme Document*. 2023 (E/ICEF/2023/P/L.22).

[czag060-B29] Vian T . Anti-corruption, transparency and accountability in health: concepts, frameworks, and approaches. Glob Health Action 2020;13:1694744. 10.1080/16549716.2019.1694744 32194010 PMC7170369

[czag060-B30] World Bank . *Malawi Economic Monitor, July 2025: Navigating Uncertainty*. 2025. https://hdl.handle.net/10986/43597 (Accessed 3 March 2026).

[czag060-B31] Zandian H, Esfandiari A, Sakha MA et al Strategies to reduce informal payments in health systems: a systematic review. East Mediterr Health J 2019;25:914–22. 10.26719/emhj.19.057 32003450

